# Electrostatic Fermentation: Molecular Response Insights for Tailored Beer Production

**DOI:** 10.3390/foods13040600

**Published:** 2024-02-16

**Authors:** Aldo Amaro-Reyes, Diana Marcial-Ramírez, Pedro Alberto Vázquez-Landaverde, José Utrilla, Monserrat Escamilla-García, Carlos Regalado, Gonzalo Macias-Bobadilla, Juan Campos-Guillén, Miguel Angel Ramos-López, Sarai E. Favela-Camacho

**Affiliations:** 1 Faculty of Chemistry, Autonomous University of Queretaro, C.U., Cerro de las Campanas S/N, Las Campanas, Querétaro 76010, QRO, Mexico; moneg14@hotmail.com (M.E.-G.); juan.campos@uaq.mx (J.C.-G.); agromyke@gmail.com (M.A.R.-L.); 2 Department of Food Research and Postgraduate Studies, Faculty of Chemistry, Autonomous University of Queretaro, C.U., Cerro de las Campanas S/N, Las Campanas, Querétaro 76010, QRO, Mexico; dianamarel325@gmail.com (D.M.-R.); regcarlos@gmail.com (C.R.); 3 Center for Research in Applied Science and Advanced Technology, Querétaro Unit, National Polytechnic Institute, Cerro Blanco 141, Colinas del Cimatario, Querétaro 76090, QRO, Mexico; pavazquez@ipn.mx; 4 Synthetic Biology Program, Center for Genomic Sciences, National Autonomous University of Mexico, Avenida Universidad 2001, Chamilpa, Cuernavaca 62210, MOR, Mexico; utrilla@ccg.unam.mx; 5 Division of Postgraduate Studies, Faculty of Engineering, Autonomous University of Querétaro, Cerro de las Campanas S/N, Las Campanas, Querétaro 76010, QRO, Mexico; gonzalo.macias@uaq.mx; 6 Institute of Engineering and Technology, Autonomous University of Ciudad Juárez, Avenida del Charro s/n y, Calle Henry Dunant, Omega, Cd Juárez 32584, CHIH, Mexico; saraifavela@hotmail.com

**Keywords:** electrostatic fermentation, *Saccharomyces pastorianus*, volatile profile, RNA-seq, low-alcohol beer

## Abstract

Electrostatic fermentation avoids the cellular redox imbalance of traditional fermentation, but knowledge gaps exist. This study explores the impact of electrostatic fermentation on the growth, volatile profile, and genetic response of *Saccharomyces pastorianus* Saflager S-23. The applied voltage (15 and 30 V) in the electrostatic fermentation system increased the growth and substrate utilization of *S. pastorianus* while decreasing ethanol production. The aromas typically associated with traditional fermentation, such as alcoholic, grape, apple, and sweet notes, were diminished, while aromas like roses, fruits, flowers, and bananas were augmented in electrostatic fermentation. RNA-seq analysis revealed upregulation of genes involved in cell wall structure, oxidoreductase activity, and iron ion binding, while genes associated with protein synthesis, growth control, homeostasis, and membrane function were downregulated under the influence of applied voltage. The electrostatic fermentation system modulates genetic responses and metabolic pathways in yeast, rendering it a promising method for tailored beer production. Demonstrating feasibility under industrial-scale and realistic conditions is crucial for advancing towards commercialization.

## 1. Introduction

Fermentation is an ancient technology used in the production and transformation of different compounds, such as biochemicals, biopharmaceuticals, biofuels, food, and beverages. However, the production yields in fermentation are limited by thermodynamics and cell regulation that maintains the metabolism in redox balance [1]. Regulating metabolic pathways in microorganisms can contribute to minimize the cost and time invested in optimizing both culture media and strain in industrial fermentations [2]. 

Electro-fermentation (EF) is a recent technology that merges traditional fermentation with electrochemistry, having the potential to open novel bio-electro production platforms to produce a wide variety of compounds through electric, electrostatic, magnetic, and electromagnetic and fields [3,4,5,6]. EF can stabilize/optimize fermentation metabolisms by controlling imbalances due to substrate purity, redox/pH conditions, and byproduct accumulation. EF may also establish oxidative or reductive conditions to drive carbon chain breakdown or elongation; increase ATP synthesis; and improve microbial biomass yield [2,7]. 

Electroactive microbes could perform bi-directional extracellular electron transfer (EET) to exchange electrons and energy with extracellular environments, thus playing a central role in microbial EF process, which opens the way to a broad range of practical biotechnological applications for the manufacture of sustainable chemicals [5,8]. Furthermore, the electric field could be used to manipulate gene expression to design biochemical networks by replacing complex biomolecular interactions with push-button operations [9]. 

Despite the ability of EF to optimize microbial processes and potentially impact emerging biomass refinery chains, there are knowledge gaps involving the impact of electrical potential and current intensity on the metabolism of the organism of interest. Additionally, the fermentations platforms are limited by the selection of microbial strains that can benefit from EET [1,6,7]. Although the earliest attempts to use electrostatic fermentation systems to promote microbial growth date back more than 50 years, the limited understanding of these phenomena hindered the scaling-up of this emerging technology [3]. 

Beer is one of the most popular alcoholic beverages across the world, typically made from malt, hops, yeast, and water, with an alcohol content ranging from 2 to 20% *v*/*v*. Recently, low-alcohol and alcohol-free beers have gained popularity as they offer a healthier alternative to alcoholic beers and can be more widely consumed [10,11]. *Saccharomyces pastorianus* is one of the world’s most important industrial organisms, renowned for its role in producing lager-style beers characterized by clean and crisp profiles. This is achieved through fermentation at lower temperatures ranging from 5 to 15 °C, which leads to reduced ester production. However, there is a growing interest in diversifying beer flavors. This includes exploring methods to introduce fruity and floral aromas, a trend that aligns with consumer preferences and current industry trends [12]. 

Additionally, aroma compounds provide attractiveness and variety to alcoholic beverages, while the variability of biosynthetic pathways activity of different yeast strains produce a dramatic effect on beer flavor [13]. Indeed, one of the main challenges when brewing a low-alcohol or alcohol-free beer is the lack of the appreciated fruitiness and the appearance of off-flavors due to the physical or biological processes applied to reduce the alcoholic content [14]. 

Gene expression is multifactorial, influenced by environmental signaling within the cell. Shedding new light on the mechanisms of gene transcription may help explain the competitive advantage of certain species and decipher the determinants of important phenotypic variation and plasticity [12,15]. Until now, brewers have relied on a relatively small number of lager yeast strains that exhibit limited phenotypic diversity. This stands in contrast to the often wide variety of strains available in other fermentation industries. Developing techniques allowing the design and creation of tailor-made production lager strains is needed [12]. 

Previous transcriptome analyses of *S. pastorianus* strains under fermentation conditions have identified differential gene expression in response to temperature and media acclimatization. These studies prove a roadmap for a more detailed transcriptomic analysis of these industrial strains [15,16].

Overall, the EF system can effectively modulate genetic responses and metabolic pathways in a microorganism, leading to improved fermentation performance and tailored compound production. This opens up exciting possibilities for exploring new, sustainable, and efficient fermentation methods for the brewing industry. This study aimed to investigate the impact of the electrostatic fermentation system on the growth, metabolism, volatile profile, and molecular responses of *Saccharomyces pastorianus* Saflager S-23.

## 2. Materials and Methods

### 2.1. Materials and Chemicals

The dry yeast *Saccharomyces pastorianus* Saflager S-23 was obtained from Lesaffre (Lille, France). Tettnanger (alpha acid 3–6% and beta acid 3–5%) and Perle (alpha acid 8–9% and beta acid 8%) German hop pellets for bittering and aroma were provided by Maltosaa (Querétaro, Mexico). Two-row spring *Hordeum distichum* Pilsener barley grains (wort color EBC 3.0–3.5) were purchased from Avangard Malz (Gelsenkirchen, Germany). Caramel 20 L barley grains (color 20 °Lovibond) were obtained from Briess Malt & Ingredients (Chilton, WI, USA). All other chemicals were sourced from Merck KGaA (St. Louis, MO, USA).

### 2.2. Brewing

The Saflager S-23 yeast (1 g) was activated in 100 mL of sterile yeast peptone dextrose (YPD) broth, which had been autoclaved at 15 lb for 15 min. The YPD broth composition was as follows: 10 g/L yeast extract, 20 g/L peptone, and 20 g/L dextrose, maintained at pH 5 and 20 °C for 24 h. Subsequently, the activated yeast was streaked onto a Petri dish containing YPD agar (broth composition plus 15 g L^−1^ agar), also at pH 5 and 20 °C, and allowed to incubate for 24 h. A single colony was then inoculated into 100 mL of YPD broth and incubated for 24 h at 150 rpm and 20 °C. Upon reaching an absorbance units of 0.6–0.8 at 600 nm (A_600_) (measured with a Genesys 10S, Thermo Fisher Scientific, Waltham, MA, USA), the cells were centrifuged at 5000× *g*, 4 °C for 15 min using a 5810R centrifuge (Eppendorf, Hamburg, Germany). The resulting pellet was then resuspended in 100 mL of wort. Barley grains were ground using a grinder (model 80350R, Hamilton Beach, VA, USA) and sieved to achieve a particle size of 600 μm. Malt extract was prepared by immersing a muslin bag containing 200 g of Pilsener and 30 g of Caramel 20 L sieved barley in one liter of filtered distilled water at 70 °C for one hour. Then, malt extract was then heated to boiling, and 0.9 g of Perle hops were added after 30 min, followed by additions of 1.3 g and 0.6 g of Tettnanger hops after 45 and 85 min of boiling, respectively. After a total boiling time of 90 min, the wort was cooled to 20 °C in an ice bath, filtered through a Nylon mesh with a pore size of 1 μm, and standardized to 8 °Brix by adding sterilized distilled water. Finally, 90 mL of the wort was inoculated with 10 mL of the resuspended yeast and transferred into the EF system.

### 2.3. Electrostatic Fermentation (EsF) System

**Figure 1:**
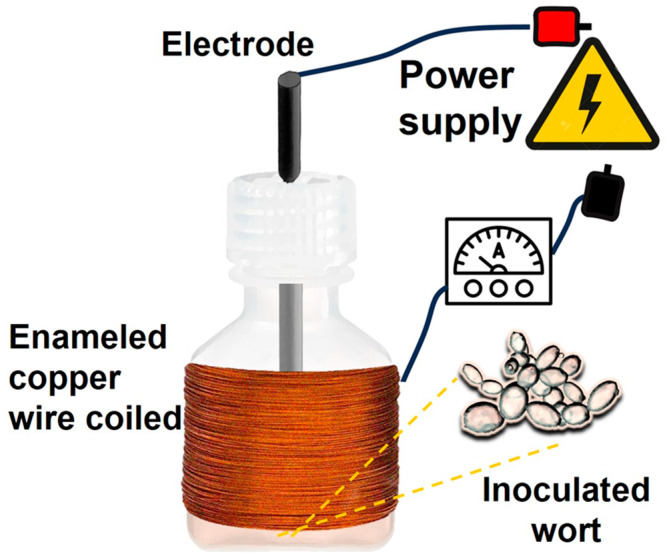
The electrostatic fermentation cell consisted of a plastic bottle with a graphite electrode located at the center of the lid and an AWG 24 enameled copper wire coiled with 300 loops around the bottle. Both the electrode and the copper wire were connected to a power supply.

The EsF system followed the methodology outlined by Mathew et al. [17], with modifications (Figure 1). The fermentation cell consisted of an electrode assembly and inoculated wort contained in a 125 mL Nalgene (Merck) polypropylene square bottle. The electrode assembly comprised a 13 cm piece of graphite positioned at the center of the bottle lid, with a 300-loop enameled copper wire (AWG 24) coiled around the bottle body. Both the electrode and the coiled copper wire were connected to a direct current voltage source (GPS-3030DD, GW Instek, New Taipei City, Taiwan), as well as to an ammeter (Mut-33, Truper, Edo. Mex, Mexico) to prevent the applied voltage from causing current in the circuit. To maintain a temperature of 16 ± 2 °C, the fermentation cells were placed in a Styrofoam cooler with ice added as necessary and monitored using a thermometer (Taylor, Rockton, IL, USA).

### 2.4. Analytical Methods and Parameter Calculations

The effect of voltage (15 and 30 V) on the growth, substrate utilization, and ethanol production of *S. pastorianus* Saflager S-23 under the EsF system was evaluated. Biomass, reducing sugars, and ethanol concentrations were quantified every 12 h for 60 h. The significant treatments were analyzed for volatile compounds identification after 60 h, and RNA-seq analysis at the middle of the exponential growth phase (24 h). A control experiment was conducted using traditional fermentation (TF) in the EsF system with no voltage applied. Biomass was determined spectrophotometrically as optical density at 600 nm (Thermo Fisher Scientific) and converted to dry cell weight (g L^−1^) using a calibration curve that relates optical density to dry biomass. The fermented wort was centrifuged (10,000× *g*, 10 min at 4 °C), and both reducing sugars and ethanol were quantified spectrophotometrically. Volatile compound identification was performed in the supernatant by solid-phase microextraction (SPME) coupled to gas chromatography/mass spectrometry (GC-MS). Reducing sugars were determined by the Dinitrosalicylic acid method (DNS) [18] with glucose as the standard, and ethanol was determined by oxidation with dichromate after a liquid–liquid extraction with tributyl phosphate [19]. The specific growth rate (μ) was determined by fitting the biomass production over time with the nonlinear Gompertz model [20], and the doubling time (td) was calculated by dividing the natural logarithm of two by the specific growth rate. Specific rates of substrate consumption (qS) and ethanol production (qEtOH) were estimated as the specific growth rate multiplied by the corresponding yield on dry biomass during the exponential phase.

### 2.5. Volatile Compound Identification

A divinylbenzene/carboxen/polydimethylsiloxane-coated fiber (DVB/CAR/PDMS 50/30 μm, Supelco, Bellefonte, PA, USA) was used for SPME. The GC-MS analysis was performed on an HP 7890A series II GC (Agilent Technologies, Wilmington, DE, USA) coupled to a mass spectrometer (HP 5975C, Agilent Technologies). Helium (99.999%) was used as the carrier gas with a column flow rate of 1.9 mL min^−1^, and the capillary column used was HP-5 (50 m × 0.32 mm inner diameter, 0.52 μm film thickness, Agilent Technologies). An MPS2 autosampler (Gerstel, Linthicum, MD, USA) was used for automatic sample feeding. The injection of the sample and the data reading were according to [21]. The presumptive identification of volatile compounds was achieved by comparing the GC retention times and mass spectra with the data system library (NIST, 2005 software, Mass Spectral Search Program V.2.0d; NIST 2005, Washington, DC, USA).

### 2.6. RNA-Seq and Bioinformatic Analyses

Total RNA for transcriptome sequencing was isolated from samples of traditional fermentation and electrostatic treatment using TRIzol (Invitrogen, Carlsbad, CA, USA), following the manufacturer’s instructions. RNA samples from two independent experiments at the same time point and treatment were evenly pooled in equal amounts and utilized for the RNA-Seq experiment. These pooled samples were then submitted to Macrogen (Seoul, Republic of Korea) for NGS transcriptome sequencing employing the Illumina HiSeq 2000 instrument with 100 bp single-end reads.

Bioinformatic analyses were performed using the Trim galore 0.6.6 program (www.bioinformatics.babraham.ac.uk accessed on 1 August 2023).) to eliminate adapters and unwanted sequences from the RNA-Seq data. The quality of the processed sequences was evaluated using the FastQC 0.11.8 (https://www.bioinformatics.babraham.ac.uk/projects/fastqc/ (accessed on 1 August 2023)) and MultiQC 1.10 (https://multiqc.info/ (accessed on 1 August 2023)) programs. Subsequently, the resulting sequences were aligned to the reference genome of *Saccharomyces pastorianus* (assembly ASM1102231v1) using the Rsubread 2.2.6 program (http://bioconductor.org/packages/Rsubread (accessed on 2 August 2023)). The reference genome was indexed as the initial step, and the samples were aligned to this index. Sample counts were acquired and normalized using the featureCounts 2.0.6 program (https://subread.sourceforge.net/featureCounts.html (accessed on 3 August 2023)) from the Rsubread package. Differential expression analysis was conducted using two distinct algorithms: the Deseq2 1.28.1 program (https://bioconductor.org/packages/release/bioc/html/DESeq2.html (accessed on 4 August 2023)) and the IDEAMEX suite [22] with filtering by a *p* value < 0.05 and fold change (FC) > 2. Significant changes in gene expression were further analyzed for functional enrichment using the UP000501346 proteome from the UniProt database (https://www.uniprot.org/ (accessed on 4 August 2023)).

### 2.7. Statistical Analysis

All experiments were performed in triplicate. The data underwent one-way analysis of variance for each experiment to determine significant differences (*p* < 0.05) among mean values, utilizing Tukey or Dunett tests with Minitab 16.2.4 (State College, PA, USA).

## 3. Results

### 3.1. Effect of Voltage on Yeast Growth, Substrate Utilization, and Ethanol Production under the Electrostatic Fermentation (EsF) System

**Figure 2:**
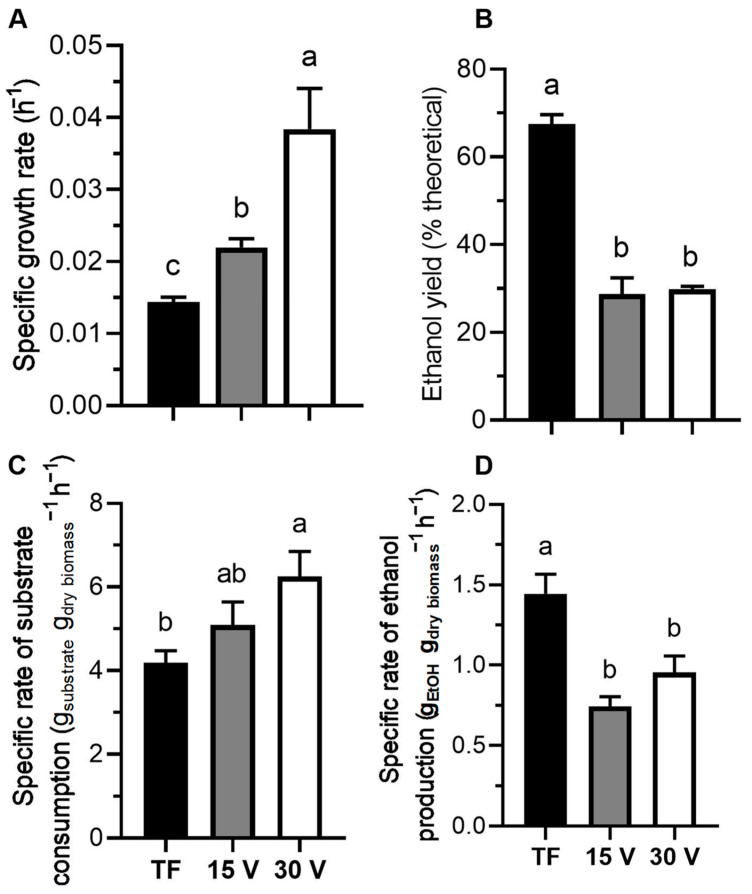
Growth and ethanol parameters of *Saccharomyces pastorianus* Saflager S-23 under the electrostatic fermentation system at 15 V (gray), 30 V (white) and traditional fermentation (TF) in black. Specific growth rate (**A**), ethanol yield (**B**), specific substrate consumption (**C**), and specific ethanol production (**D**). Different letters indicate statistical significance (*p* < 0.05). Results are reported as the mean ± standard deviation (*n* = 3).

The applied voltage in the EsF system exhibited significant differences (*p* < 0.05) in the growth and substrate utilization of *Saccharomyces pastorianus* Saflager S-23. Compared to traditional fermentation (TF), ethanol production decreased (Figure 2). The specific growth rate of *S. pastorianus* using 30 V (0.0384 h^−1^) was 1.7- and 2.7-fold higher than in the treatments at 15 V and TF, respectively. The doubling time decreased by 34.4% and 62.5% when the graphite electrode was used at 15 V and 30 V, respectively, compared to the 48.1 h obtained in TF. Ethanol yield significantly decreased to around 22.5% and 29.3% when the graphite electrode and voltage were applied (15 V and 30 V, respectively), compared to TF (67.5% of the maximum theoretical). The specific rate of substrate consumption (6.3 g_substrate_ g_dry biomass_^−1^ h^−1^) was up to 1.7-fold higher when applying 30 V and the graphite electrode than TF. The specific rate of ethanol production reached a maximum value of 1.4 g EtOH g_dry biomass_^−1^ h^−1^ in TF, and it decreased by 48.5% and 33.9% in 15 V and 30 V, respectively. It is evident that the application of voltage in an electrostatic system modulates the metabolism of *Saccharomyces pastorianus* Saflager S-23.

### 3.2. Volatile Compound Identification

**Figure 3:**
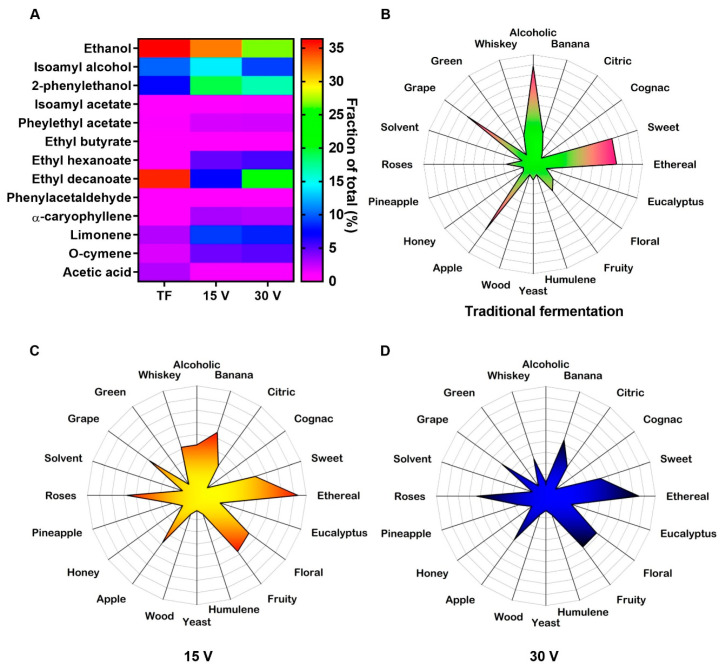
Volatile compound identification on the wort at 60 h in traditional fermentation (TF), 15 V, and 30 V (**A**). Aroma profile of traditional fermentation (**B**), and in the electrostatic fermentation system applying 15 V (**C**) and 30 V (**D**). Lighter colors correspond to lower concentrations, while darker colors correspond to higher concentrations.

As the applied voltage produced differences in the yeast growth, the volatile compounds in beer could also be affected by the EsF system. The HS-SPME GC-MS analysis was conducted on the wort at 60 h in traditional fermentation, 15 V, and 30 V (Figure 3A). More than 63 volatile compounds were identified in all treatments, of which 85% were shared. A selection of specific volatile compounds closely related to lager beers were identified, and for most of the volatiles detected, the relative concentration was higher when applying voltage (15 V and 30 V) than using TF. The higher alcohols, such as isoamyl alcohol and 2-phenylethanol, the ester ethyl hexanoate, and the terpenes caryophyllene, limonene, and o-cymene, showed 1.4- to 7.3-fold higher relative concentrations than TF. However, EsF produced a lower concentration of ethanol, ethyl decanoate, and acetic acid than TF, as shown in the ethanol production parameters. In contrast, ethyl hexanoate was only detected when voltage was applied. The aroma profile of TF (Figure 3B) was modified by applying 15 V and 30 V (Figure 3C,D). The alcoholic, grape, apple, and sweet aromas of traditional fermentation were diminished, while those of roses, fruits, flowers, and bananas were augmented after EsF.

### 3.3. RNA-Seq Analyses

**Figure 4:**
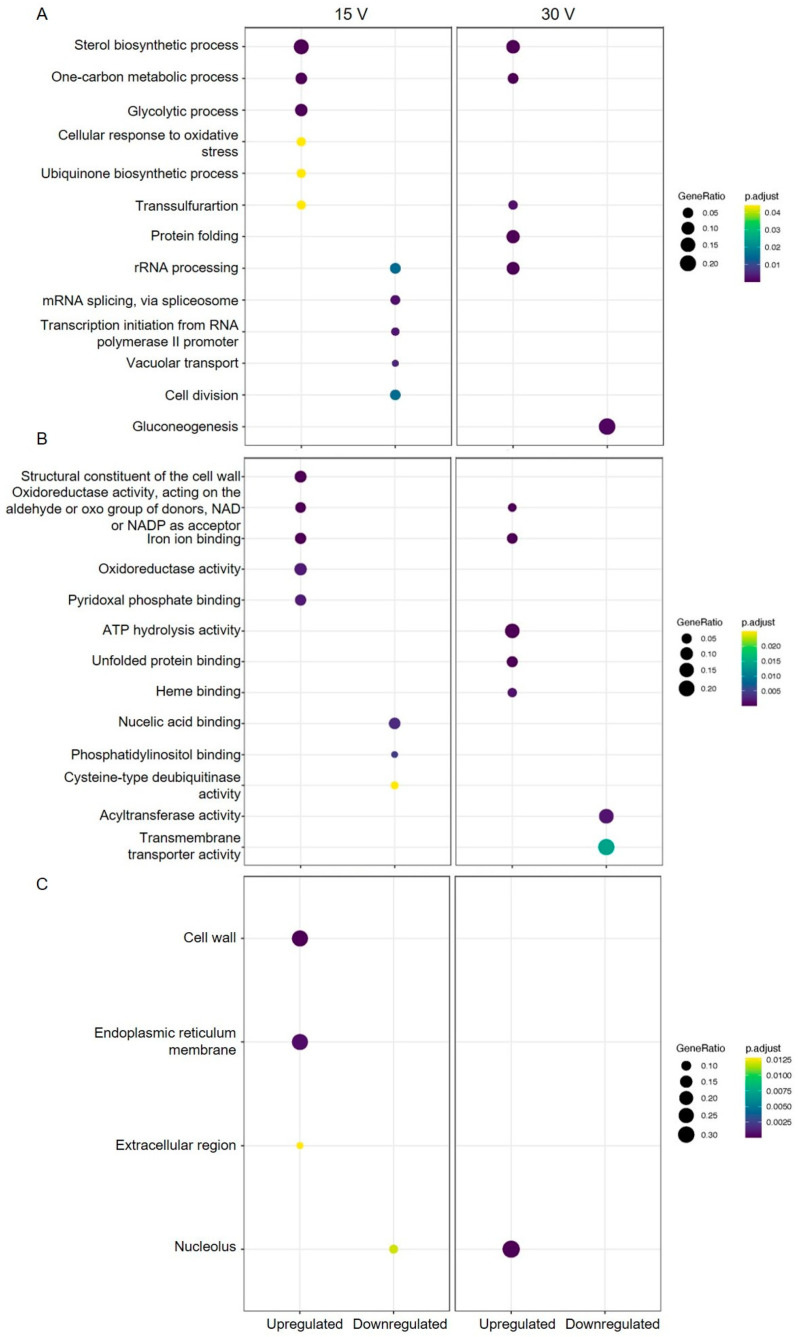
Gene ontology enrichment comparison of differentially expressed genes for biological process (**A**), molecular functions (**B**), and cellular components (**C**) in the electrostatic fermentation system applying 15 V and 30 V compared to traditional fermentation (*p* < 0.05 and fold change >2).

The RNA-sequences were mapped to the genome of *Saccharomyces pastorianus* (assembly ASM1102231v1) after removing the adapters and non-desired sequences, to correlate the normalized samples counts. Then, a gene enrichment analysis (FC > 2, *p* < 0.05) was performed on the differentially expressed genes (DEGs) from the TF versus EsF for 15 V and 30 V, using the proteome UP000501346 (https://www.uniprot.org accessed on 5th August 2023). The expression profiles for biological process, molecular functions, and cellular components showed that at 15 V, 13 genes were significantly overexpressed and nine were downregulated. Meanwhile, at 30 V, 11 genes were upregulated and three where downregulated (Figure 4). Out of the total DEGs, only five were upregulated and two were downregulated in both conditions of the EsF system. Surprisingly, the 15 V condition exhibited a larger transcriptional response than 30 V condition, indicating a non-linear correlation between applied voltages in EsF system and the molecular effects.

The DGEs in the biological processes of *S. pastorianus* that exhibited upregulation at both 15 V and 30 V were those involved in the sterol biosynthetic process, the one-carbon metabolic process, and transsulfuration. Additionally, among the upregulated genes, those related to the cellular response to oxidative stress and the ubiquinone biosynthetic process were identified at 15 V (Figure 4A). Conversely, the genes associated with protein folding and rRNA processing were also found to be upregulated at 30 V. In contrast, when using the EsF system at 15 V, a downregulation of genes associated with rRNA processing, mRNA splicing, via spliceosome, transcription initiation from RNA polymerase II promoter, vacuolar transport, and cell division was observed. Moreover, at 30 V, only gluconeogenesis genes showed downregulation. Remarkably, the rRNA processing genes displayed an inverse response to the applied voltages. While the downregulation response affected a greater number of genes at 15 V, the gen ratio response was even more pronounced at 30 V (Figure 4A).

Conversely, genes associated with the molecular function of *S. pastorianus* exhibited upregulation in response to the influence of the EsF system at both 15 V and 30 V, particularly those engaged in oxidoreductase activity involving the aldehyde or oxo group of donors, utilizing NAD or NADP as acceptors, as well as iron ion binding. At 15 V, genes linked to the structural constituent of the cell wall, oxidoreductase activity, and pyridoxal phosphate binding were upregulated. However, at 30 V, the upregulated genes were predominantly related to ATP hydrolysis activity, unfolded protein binding, and heme binding (Figure 4B). Furthermore, under the influence of the EsF system at 15 V, there was downregulation observed in genes associated with nucleic acid binding, phosphatidylinositol binding, and cysteine-type deubiquitinase activity. In contrast, at 30 V, the downregulation effect was more pronounced among genes involved in acyltransferase activity and transmembrane transporter activity, surpassing the response observed at 15 V in terms of gene ratio (Figure 4B).

Regarding the DEGs related to cellular components, the upregulated genes at 15 V were associated with the cell wall, endoplasmic reticulum membrane, and the extracellular region. Additionally, the downregulated genes at 15 V were related to the nucleolus. However, at 30 V, these genes were upregulated, and no genes showed downregulation (Figure 4C).

## 4. Discussion

This research contributes to our understanding of the molecular mechanism, growth variations, and volatile profile induced by an EsF system. This work found faster growth and substrate consumption in *Saccharomyces pastorianus* Saflager S-23 under the EsF system in agreement with previous observations [17] using *S. cerevisiae*. *S. pastorianus* displayed significant differences in specific growth rate, doubling time, and the specific rate of ethanol production compared to TF. These findings provide evidence of its ability to use graphite electrodes as donors/acceptors of electrons for substrate oxidation/reduction. Beretta et al. [3] and Schievano et al. [7] have reported similar electrochemical activities in different microorganisms, supporting the idea that they interact with the electrodes to facilitate electron transfer during fermentation. These electroactive microorganisms may interact through quorum sensing via low-molecular-weight sensor molecules, outer-membrane vesicles, membranous nanotubes, type IV pili, cytochrome nanowires, and small diffusible metabolites such as hydrogen, formate, and flavins [8]. 

It has been demonstrated that EsF accelerates glucose fermentation in *Saccharomyces cerevisiae*, leading to ethanol production without consuming external energy. The applied voltage could create an electric field within the cell, thereby accelerating cellular electron transport and ultimately enhancing the fermentation rate. However, more studies with well-defined yeast genotypes are needed to elucidate the molecular mechanism and the effect on the fermentation broth using an EsF system [17]. 

Furthermore, electrostatic field impacts the Debye screening, altering the behavior of charged particles through dielectrophoresis [9], thus disturbing the electrostatic interactions of cell biomolecules and modulating the metabolism at the genetic level, as evidenced in this work. The impact of the electrostatic system on the growth, metabolism and volatile profile of *S. pastorianus* could be attributed to the downregulation of genes involved in rRNA processing, mRNA splicing, via spliceosome, transcription initiation from RNA polymerase II promoter, vacuolar transport, cell division, gluconeogenesis, nucleic acid binding, cysteine-type deubiquitinase activity and nucleolus. This effect can lead to altered gene expression patterns, affecting the synthesis of essential proteins [9,23,24], as well as biomolecules storage and the growth control [25], thereby disrupting the biological process of the yeast. Moreover, *S. pastorianus* exhibits significant upregulation in genes associated with the one-carbon metabolic process, transsulfuration, the ubiquinone biosynthetic process, protein folding and rRNA processing, endoplasmic reticulum membrane, glycolytic process, ATPase activity, protein folding, and unfolded protein binding. These genetic responses under the EsF system reflect the ability of the yeast to fine-tune its cellular machinery and biochemical pathways, resulting in changes in fermentation efficiency and metabolite production [26,27,28]. The coordinated regulation of these genes highlights the adaptive nature of the Saflager S-23 yeast in response to the EsF conditions.

On the other hand, electrostatic fields have been shown to impact cell physiology and morphology, as well as the chemical–physical characteristics of the cellular membrane, membrane permeability and potential [29,30]. These effects may be explained by a possible molecular mechanism related to the downregulation of genes associated with vacuolar transport and extracellular region, disrupting cellular sorting and trafficking as well as cell signaling, leading to intra- and extra-cellular homeostasis disruptions [25], as shown in *S. pastorianus*. Additionally, the downregulation of genes associated with phosphatidylinositol binding, transmembrane transporters, and acyl group transferase activity, due to the EsF system, can affect cell signaling, membrane dynamics and composition. This in turn, can hinder the import and export of crucial molecules, influencing essential processes such as membrane trafficking and remodeling, as well as causing imbalances in nutrient uptake and waste removal and affecting membrane structure and function [31,32]. In response to these unique effects, cells may exhibit an upregulation of genes involved in the biosynthetic process of sterols, which are key constituents of the plasma membrane and structural components of the cell wall [33]. This genetic response is likely a protective mechanism against the reported stress conditions induced by the electrostatic field, such as extreme pH values, toxic species, or radicals [3,7]. The difference in gene expression related to cell physiology and molecular responses is reflected in the observed variations in growth and volatile patterns in the Saflager S-23 yeast and is consistent with previous work [16]. While hundreds of aroma-active compounds have been found in beer, reviews tend to focus mainly on alcohols and esters produced by brewing [13], as in this work. Furthermore, the increase in NADH availability resulting from the enhanced expression of oxidoreductase activity genes in *S. pastorianus* leads to different patterns of redox homeostasis [28,34]. Thus, the metabolic networks, as observed in the different patterns of growth, substrate consumption, ethanol production, and volatile compounds using the EsF system. In *S. cerevisiae*, iron plays a vital role in regulating oxidative stress and metabolic remodeling, enabling the yeast to thrive under oxidative stress [35]. Consequently, in response to the reactive oxygen species or hydroxyl radicals generated by the EsF system, the genes involved in cellular response to oxidative stress, iron and heme binding were upregulated.

Surprisingly, decreased ethanol production was observed in Saflager S-23 using the EsF system, which suggests an alternative method for producing low-alcohol beer. The low and nonalcoholic beer industry, valued at over $102 million only in the United States, offers a new image for these beverages and has the potential to attract new categories of consumers, such as women and teenagers, who can exploit their health benefits without the risk of alcohol intake [10,11]. Finally, fruity and floral aromas are in high demand in the beverage industry, and continuous efforts are being made to enhance the beer aroma by increasing or diversifying the fruity flavor profile [13]. Thus, the proposed EsF system could serve as an alternative for tailored beer production or the creation of new health and wellness beverages. It is important to note that the chosen fermentation process does not aim to replicate the complete fermentation process of traditional lager brewing. Instead, it seeks to investigate specific aspects of yeast behavior and fermentation performance within the context of the electrostatic fermentation system. Future studies may indeed explore lager fermentation dynamics and the influence of mineral content on beer quality. In addition to HS-SPME analysis, conducting sensory panels to assess attributes such as aroma, taste, mouthfeel, and overall drinkability will contribute to confirming that the beer retains its desired characteristics and fulfills the requirements of beer quality.

The advancement of electrofermentation represents a promising frontier in fermentation science, yet realizing its full potential necessitates robust experimental evidence across various aspects, including reactor design, energy optimization, and cost–benefit analysis. Demonstrating feasibility on a laboratory scale under realistic conditions is pivotal for progressing toward pilot studies, scale-up, and eventual commercialization. By addressing these challenges, the path to leveraging electrofermentation for enhanced fermentation efficiency and product quality while ensuring cost-effectiveness and practical feasibility becomes clearer, paving the way for significant advancements in fermentation technology.

## 5. Conclusions

*Saccharomyces pastorianus* Saflager S-23 effectively engages with graphite electrodes in an EsF setup, revealing a complex link between genetic responses and metabolic pathways during electron transfer in fermentation. This EsF approach influences cellular processes, metabolic pathways, membrane integrity, and function, safeguarding *S. pastorianus* against stress. This defense yields varied fermentation outcomes and efficiencies. The EsF technique holds great promise for customized beer production, exhibiting potential in shaping yeast metabolic networks for industrial usage. Grasping EsF’s impact on yeast physiology and fermentation will guide future research in this burgeoning biotechnology realm, opening doors for advanced exploration. Although laboratory-scale EF systems have been developed, challenges such as identifying and producing high-quality products, developing efficient reactors, uncovering electron transfer mechanisms, and reducing costs persist. Innovations in material development, electrode design, metabolic engineering, synthetic biology, and fermentation techniques are underway, but multidisciplinary efforts are needed. Optimizing reactor design, refining energy input, conducting thorough cost–benefit analyses, and demonstrating feasibility under realistic conditions are crucial for advancing towards pilot studies and eventual commercialization.

## Data Availability

Data is contained within the article.

## References

[1] Moscoviz R., Toledo-Alarcón J., Trably E., Bernet N. (2016). Electro-fermentation: How to drive fermentation using electrochemical systems. Trends Biotechnol..

[2] Bhagchandanii D.D., Babu R.P., Sonawane J.M., Khanna N., Pandit S., Jadhav D.A., Khilari S., Prasad R. (2020). A comprehensive understanding of electro-fermentation. Fermentation.

[3] Beretta G., Mastorgio A.F., Pedrali L., Saponaro S., Sezenna E. (2019). The effects of electric, magnetic and electromagnetic fields on microorganisms in the perspective of bioremediation. Rev. Environ. Sci. Biotechnol..

[4] Chung T.H., Dhar B.R. (2021). An overview of electro-fermentation as a platform for future biorefineries. Sustain. Solut. Environ. Pollut..

[5] Gong Z., Yu H., Zhang J., Li F., Song H. (2020). Microbial electro-fermentation for synthesis of chemicals and biofuels driven by bi-directional extracellular electron transfer. Synth. Syst. Biotechnol..

[6] Kracke F., Lai B., Yu S., Krömer J.O. (2018). Balancing cellular redox metabolism in microbial electrosynthesis and electro fermentation—A chance for metabolic engineering. Metab. Eng..

[7] Schievano A., Pepé Sciarria T., Vanbroekhoven K., De Wever H., Puig S., Andersen S.J., Rabaey K., Pant D. (2016). Electro-fermentation—Merging electrochemistry with fermentation in industrial applications. Trends Biotechnol..

[8] Paquete C.M., Rosenbaum M.A., Bañeras L., Rotaru A.E., Puig S. (2022). Let’s chat: Communication between electroactive microorganisms. Bioresour. Technol..

[9] Efrat Y., Tayar A.M., Daube S.S., Levy M., Bar-Ziv R.H. (2018). Electric-field manipulation of a compartmentalized cell-free gene expression reaction. ACS Synth. Biol..

[10] Baiano A. (2021). Craft beer: An overview. Compr. Rev. Food Sci. Food Saf..

[11] Sancén M., Léniz A., Macarulla M.T., González M., Milton-Laskibar I., Portillo M.P. (2023). Features of non-alcoholic beer on cardiovascular biomarkers. Can it be a substitute for conventional beer?. Nutrients.

[12] Gibson B., Liti G. (2015). *Saccharomyces pastorianus*: Genomic insights inspiring innovation for industry. Yeast.

[13] Holt S., Miks M.H., De Carvalho B.T., Foulquié-Moreno M.R., Thevelein J.M. (2019). The molecular biology of fruity and floral aromas in beer and other alcoholic beverages. FEMS Microbiol. Rev..

[14] Piornos J.A., Koussissi E., Balagiannis D.P., Brouwer E., Parker J.K. (2023). Alcohol-free and low-alcohol beers: Aroma chemistry and sensory characteristics. Compr. Rev. Food Sci. Food Saf..

[15] Timouma S., Balarezo-Cisneros L.N., Pinto J., De La Cerda R., Bond U., Schwartz J.M., Delneri D. (2021). Transcriptional profile of the industrial hybrid *Saccharomyces pastorianus* reveals temperature-dependent allele expression bias and preferential orthologous protein assemblies. Mol. Biol. Evol..

[16] de la Cerda-Garcia Caro R., Hokamp K., Roche F., Thompson G., Timouma S., Delneri D., Bond U. (2022). Aneuploidy influences the gene expression profiles in *Saccharomyces pastorianus* group I and II strains during fermentation. PLoS Genet..

[17] Mathew A.S., Wang J., Luo J., Yau S.T. (2015). Enhanced ethanol production via electrostatically accelerated fermentation of glucose using *Saccharomyces cerevisiae*. Sci. Rep..

[18] Miller G.L. (1959). Use of dinitrosalicylic acid reagent for determination of reducing sugar. Anal. Chem..

[19] Seo H.B., Kim H.J., Lee O.K., Ha J.H., Lee H.Y., Jung K.H. (2009). Measurement of ethanol concentration using solvent extraction and dichromate oxidation and its application to bioethanol production process. J. Ind. Microbiol. Biotechnol..

[20] Tjørve KM C., Tjørve E. (2017). The use of Gompertz models in growth analyses, and new Gompertz-model approach: An addition to the Unified-Richards family. PLoS ONE.

[21] Vazquez-Landaverde P.A., Qian M.C. (2007). Antioxidant impacts on volatile formation in high-pressure-processed milk. J. Agric. Food Chem..

[22] Jimenez-Jacinto V., Sanchez-Flores A., Vega-Alvarado L. (2019). Integrative Differential expression analysis for multiple experiments (IDEAMEX): A web server tool for integrated RNA-seq data analysis. Front. Genet..

[23] Chen F.X., Smith E.R., Shilatifard A. (2018). Born to run: Control of transcription elongation by RNA polymerase II. Nat. Rev. Mol. Cell Biol..

[24] Shenasa H., Bentley D.L. (2023). Pre-mRNA splicing and its cotranscriptional connections. Trends Genet..

[25] Attfield P.V. (2022). Crucial aspects of metabolism and cell biology relating to industrial production and processing of Saccharomyces biomass. Crit. Rev. Biotechnol..

[26] Lao-Martil D., Schmitz JP J., Teusink B., van Riel NA W. (2023). Elucidating yeast glycolytic dynamics at steady state growth and glucose pulses through kinetic metabolic modeling. Metab. Eng..

[27] Qin N., Li L., Ji X., Pereira R., Chen Y., Yin S., Li C., Wan X., Qiu D., Nielsen J. (2023). Flux regulation through glycolysis and respiration is balanced by inositol pyrophosphates in yeast. Cell.

[28] Xiao C., Pan Y., Huang M. (2023). Advances in the dynamic control of metabolic pathways in Saccharomyces cerevisiae. Eng. Microbiol..

[29] Lewis T.A., Rodriguez R.J., Parks L.W. (1987). Relationship between intracellular sterol content and sterol esterification and hydrolysis in Saccharomyces cerevisiae. Biochim. Biophys. Acta (BBA)/Lipids Lipid Metab..

[30] Ma B.X., Ke X., Tang X.L., Zheng R.C., Zheng Y.G. (2018). Rate-limiting steps in the *Saccharomyces cerevisiae* ergosterol pathway: Towards improved ergosta-5,7-dien-3β-ol accumulation by metabolic engineering. World J. Microbiol. Biotechnol..

[31] Aguilera-Romero A., Lucena R., Sabido-Bozo S., Muñiz M. (2023). Impact of sphingolipids on protein membrane trafficking. Biochim. Biophys. Acta-Mol. Cell Biol. Lipids.

[32] Bailoni E., Partipilo M., Coenradij J., Grundel DA J., Slotboom D.J., Poolman B. (2023). Minimal out-of-equilibrium metabolism for synthetic cells: A membrane perspective. ACS Synth. Biol..

[33] Subczynski W.K., Pasenkiewicz-Gierula M., Widomska J., Mainali L., Raguz M. (2017). High cholesterol/Low cholesterol: Effects in biological membranes: A review. Cell Biochem. Biophys..

[34] Hou J., Scalcinati G., Oldiges M., Vemuri G.N. (2010). Metabolic impact of increased NADH availability in Saccharomyces cerevisiae. Appl. Environ. Microbiol..

[35] Matsuo R., Mizobuchi S., Nakashima M., Miki K., Ayusawa D., Fujii M. (2017). Central roles of iron in the regulation of oxidative stress in the yeast Saccharomyces cerevisiae. Curr. Genet..

